# Association of periodontal disease with gestational diabetes mellitus among postpartum women at a private tertiary care hospital of Karachi, Pakistan: a cross-sectional study

**DOI:** 10.1038/s41598-024-60659-6

**Published:** 2024-04-30

**Authors:** Wafa Zehra Jamal, Farhan Raza Khan, Nadeem Zuberi, Syed Murtaza Raza Kazmi, Shafquat Rozi

**Affiliations:** 1 Department of Community Health Sciences, Aga Khan University, Karachi, Pakistan; 2 Department of Surgery, Aga Khan University, Karachi, Pakistan; 3 Department of Obstetrics & Gynecology, Aga Khan University, Karachi, Pakistan; 4 Department of Surgery, Aga Khan University, Karachi, Pakistan

**Keywords:** Gestational diabetes, Periodontal disease, Pregnancy, Periodontitis, Gingivitis, Dental diseases, Gestational diabetes

## Abstract

Due to the overlapping aetiology of Gestational Diabetes Mellitus (GDM) and Periodontal disease (PD), which are prevalent metabolic disorder and chronic inflammatory disorder in pregnant women respectively, they are often at risk of developing both diseases simultaneously. This study aims to evaluate the association of periodontal disease and gestational diabetes mellitus among post-partum women who delivered within 24 h at private tertiary care hospital, Karachi, Pakistan. Analytical cross sectional study with sample size of 178 by non- probability purposive sampling, a total of 101 postpartum women (57%) were diagnosed with periodontal disease and 50 (28%) were GDM positive. Of those who had PD, 35% (n = 35/101) were GDM positive. An insignificant association of the prevalence ratio of GDM in women with periodontal disease was found. [PR = 1.7; 95% CI: 0.2–3.2; *p*-value 0.07] A statistically significant association was found between the prevalence ratio of GDM in women with obesity. It was 2.6 times compared to women who were not obese (*p* value < 0.01, 95% CI: 1.3–5.1). There is insignificant association found between the prevalence ratio of GDM in women with periodontal disease in our setting. Women who are overweight or tend to gain weight should be closely monitored and guided to take dietary measures.

## Introduction

Glucose or carbohydrate intolerance of varying degree, with new onset or recognition during pregnancy is termed as gestational diabetes mellitus (GDM)^1^. This type of hyperglycaemia in pregnancy is not expected to persist postpartum^2^. It is one of the most common metabolic disorder of pregnancy with an increasing incidence and a global prevalence ranging from 1 to 45% in pregnant women^3^. The varying prevalence reflects the dependence on the characteristics of the population and the methods used for screening and diagnosing. It is estimated, globally, one in six live births are to women with some form hyperglycaemia in pregnancy out of which majority (84%) are due to GDM^4^. Globally, the South East Asian region has the highest prevalence of GDM (27%) and live births effected by GDM^5^. A higher prevalence is seen in the low-middle-income countries (LMIC) where access to maternal care is often inadequate^5^. A study reported prevalence of 13.9% GDM cases among pregnant women in the South Asian region^6^. The reported prevalence of GDM in Pakistan has increased from 3.3% in 1996 to 17.2% in 2017^7,8^. GDM is a significant cause of perinatal morbidity with adverse birth outcomes including macrosomia, maternal cardiovascular disorders and subsequent high risk of type 2 diabetes and obesity in both mother and baby^9–11^.

Periodontal disease (PD) is one of the most common chronic inflammatory disorders affecting the tissues surrounding the teeth and has a global prevalence of about 20–50% in the adult population^12^. It is characterized by one or more of the conditions; accumulation of plaque and calculus around the tooth surface near the gums, bleeding on probing the gums, attachment loss, recession and bone loss. The global prevalence of periodontal disease among pregnant women is reported to vary from 11 to 100%^13^. A study from Pakistan reported prevalence ranging from 56 to 87% of varying periodontal conditions among pregnant women^14^. Another study from a province in Pakistan reported prevalence ranging from 21 to 79% in female patients from dental hospitals^15^. The periodontal disease, as a source of sub-clinical and persistent infection, may induce systemic inflammatory responses that may result in complications during pregnancy (low birth weight, still birth, preeclampsia, anomaly etc.). Epidemiological studies of periodontal disease among pregnant women have observed association with poor pregnancy outcomes^13,14,16^. Literature highlights an association between periodontal disease and adverse pregnancy outcomes, such as preterm birth and low birth weight^17^.

The disease progression of both the conditions is affected by multiple factors. It has been postulated that few risk factors are common to the development of both GDM and periodontal disease such as age of the individual, sedentary lifestyle, poor dietary habits and obesity. Therefore, due to overlapping aetiology, a person exposed to these risk factors is at risk of developing both diseases simultaneously. Studies exploring the association of GDM and periodontal disease are limited and reported to have found a variable relationship between the two conditions. Few studies have explored the co-existence of GDM and periodontal disease. A study from USA found a high prevalence (77.4%) of periodontal disease among GDM patients^18^. Whereas a study conducted in India reported no meaningful association between the two conditions^6^. Studies from Indonesia, West Africa, Sudan, Pakistan and Brazil have also provided some evidence of association between pregnancy and periodontal disease^19–23^. However, varying results demand a greater need of further studies to explore the relationship with better study designs for a meaningful impact since both the conditions can be prevented and treated successfully if diagnosed timely.

Given the high burden of both GDM and periodontal disease in Pakistan it is imperative to explore the relationship between these two diseases. Both the conditions are known to cause maternal and infant morbidity which already has a high burden in Pakistan. Exploring the relationship will fill the knowledge gap that can help identify the need to take timely measures to prevent both periodontal disease and GDM as both can be easily diagnosed and treated early to avoid unfavourable outcomes, especially in low resource countries such as Pakistan where a dental check-up is not a part of regular antenatal care. The study aims to evaluate the association of periodontal disease and gestational diabetes mellitus (GDM) among post-partum women at private tertiary care hospital of Karachi, Pakistan. The study hypothesis is that the prevalence ratio of GDM in women with periodontal disease is greater as compared to women without periodontal disease.

## Materials and methods

### Study design and setting

This is an analytical cross sectional study design to infer the association between periodontal disease and GDM. Data is collected from women delivering babies (postpartum) within 24 h at private tertiary care hospital, Aga Khan university hospital of Karachi, Pakistan.

### Eligibility criteria

#### Inclusion criteria

i. Postpartum women who delivered babies within the last 24 h at AKUH.
ii. Women without any prior history of existing diabetes
iii. Those giving consent for participation.

#### Exclusion criteria

i. Altered Glasgow Coma Score of postpartum women. (those women may not be in the condition to participate)
ii. Women with metabolic bone disease. (those women might already be suffering from periodontal disease due to bone disorder)
iii. Women taking calcium channel blockers, phenytoin, immunosuppressant and anticonvulsants (medication that causes gingival hyperplasia)
iv. More than 8 teeth missing (may include index teeth that need to be examined, or may be using dental prosthesis for missing teeth that may affect periodontal health)

### Approach to study participants

Permission from the relevant department (Gynecology and Obstetrics ward) was sought prior to the initiation of data collection process. Daily list of deliveries was readily available in the respective department and was provided to the data collectors on request. Patients were enrolled from the post-partum ward using non-probability purposive sampling method. All the participants were administered a screening questionnaire to check for eligibility criteria. In addition, the eligibility criteria were also cross-checked from the patient’s medical record. A written informed consent was taken from all the eligible participants after explaining them the study objectives and mechanics in detail. Data collection took place from July 2022 till September 2022.

A structured questionnaire was used to collect socio-demographic and disease related information from each patient through interview and medical record review. The questionnaire included tools that are validated to measure GDM and periodontal disease. A pilot test of the questionnaire was conducted on 5% of the sample size at a similar setting, which lead to no major revisions in the questionnaire, however, due to unavailability of pre-pregnancy weight, a criterion of using 1^st^ trimester weight for BMI was set.

Exposure assessment for the presence of periodontal disease was carried out using a structured questionnaire and physical examination. Data collectors were Bachelor of Dental Surgery, graduate dentists, received a 3 day training as per the instructions in expert approved manual of operations prior data collection process to ensure reliability. The dental examination was carried out under standard operating procedures (SOPs) at the participants own bed or chairside. The dentist (data collector) was wearing clean utility gloves, protective eyewear, facemask and lab coat while performing the examination. Examination was done using sterilized mouth mirror and a periodontal probe (UNC15).

Non- probability purposive sampling was used to enroll the study participants. Sample size was calculated using Open-epi software^24^. Percent of exposed (50% with periodontal disease) and unexposed (26% without periodontal disease) with the outcome (GDM) were taken from the reference study^25^. Keeping 5% level of significance, 80% power and adjusting for 10% non-response rate a sample size of 157 was calculated. This was the minimum required sample size. However, we achieved a sample size of 178 in this study.

### Variables of interest

#### Outcome variable: gestational diabetes status

GDM status of the postpartum women was obtained through the structured questionnaire and confirmed from medical records. It is a routine practice to screen every pregnant woman for GDM using the Oral Glucose Tolerance Test (OGTT). The diagnosis of GDM was completed by a 2 h oral glucose tolerance test (OGTT) performed with 75 g of glucose, according to the International Association of Diabetes and Pregnancy Study Group criteria^26^. The results of the OGTT are readily available in patient’s file.

#### Exposure variable: periodontal disease (includes both gingivitis and periodontitis)

Probing Depth (PD), Clinical Attachment Loss (CAL) and Bleeding on Probing (BOP) was noted for four sides of index teeth. Periodontitis is defined as the presence of ≥ 4 teeth having ≥ 1 sites with PD ≥ 4 mm and CAL ≥ 3 mm associated with BOP^27,28^. Gingival index is determined as—0: normal, 1: mild inflammation slight color change and edema and no bleeding on probing, 2: moderate inflammation redness, edema with bleeding on probing, 3: severe inflammation, marked redness and edema, ulceration and spontaneous bleeding. Total Gingival Index (GI) score was calculated as total score divided by number of surfaces examined. As per the GI score, gingivitis is classified as—mild (0.1 − 1), moderate (1.1− 2) and severe gingivitis (2.1− 3)^29^.

### Other variables

BMI was categorized as 30kg/m^2^ and above as obese, 25.0–29.9 kg/m^2^ as overweight and 18.5–24.9 kg/m^2^ as normal or healthy weight. Birthweight less than 2500 g was termed as low birth weight. Gestational age less than 36 weeks was labelled as Preterm.

A screening questionnaire, based on eligibility criteria was used after which a structured questionnaire was employed which included examination for periodontal disease.

### Data management

Principal investigator and data entry officers entered the data separately, both datasets were compared for consistency & missing values. Data entry was done on Excel and transferred on Stata version 17.0 for cleaning and editing. Editing was done on a daily basis to ensure there are no missing values, no illogical or inappropriate entries, completeness and accuracy of data. Missing values or inappropriate information were rechecked for correction and verification. Cleaning of data was carried out to sort responses in ascending order and data is sequenced in a logical manner. The link between ID # and name is kept secured and confidential. Electronic data is secured with a strong password and data will be discarded 5 years after the study as per protocol.

### Statistical analysis

Gestational diabetes as a binary categorical outcome and periodontal disease as binary categorical exposure variable were examined. Data analysis is performed on Stata version 17.

For a descriptive analysis, dispersion and distribution of quantitative variables was seen. For normal distribution of quantitative variables mean and standard deviation is reported. Proportions of characteristics, exposures and outcome of qualitative variables is tabulated. Frequency and percentages is seen for ordinal and normal distribution of categorical data. Chi square test was used to examine the association between two categorical variables and independent t test is applied to compare the means of two groups of continuous numerical data.

Cox Proportional hazard regression is used to obtain unadjusted and adjusted prevalence ratios with 95% confidence interval and p value, in a step wise model building approach.

Each variable is regressed independently with outcome variable (GDM), their assumptions checked and significance reported. Multicollinearity, interaction and confounding is assessed individually using the appropriate tests. The Multicollinearity was assessed by using coefficient of correlation. The Pearson correlation was used in normally quantitative distributed variables. The Eta correlation was used to assess the correlation between one qualitative and one quantitative explanatory variable. The Cramer’s V for correlation between two qualitative explanatory variables.

### Ethical approval and considerations

This study received approval from the Ethics Review Committee, Aga Khan University Hospital (2022–7086-21752). The study was conducted in accordance with the Helsinki Declaration, revised in 2013. An informed consent from every participant was taken, where they were made aware of the study implications, its importance and their right to refuse to participate without any consequence. Confidentiality was maintained at every step. The research poses no more than minimal risks to participants and does not give any rise to the disclosure of the participant’s identity. In addition, the analysis has an important public health function. A referral form was provided to those who were diagnosed/ suspected with either periodontal disease or gestational diabetes for their management and treatment. An educational/informational pamphlet was provided to all participants to create awareness and knowledge. Each woman was counselled about the condition of her oral health and referred for treatment if needed.

## Results

**Figure 1:**
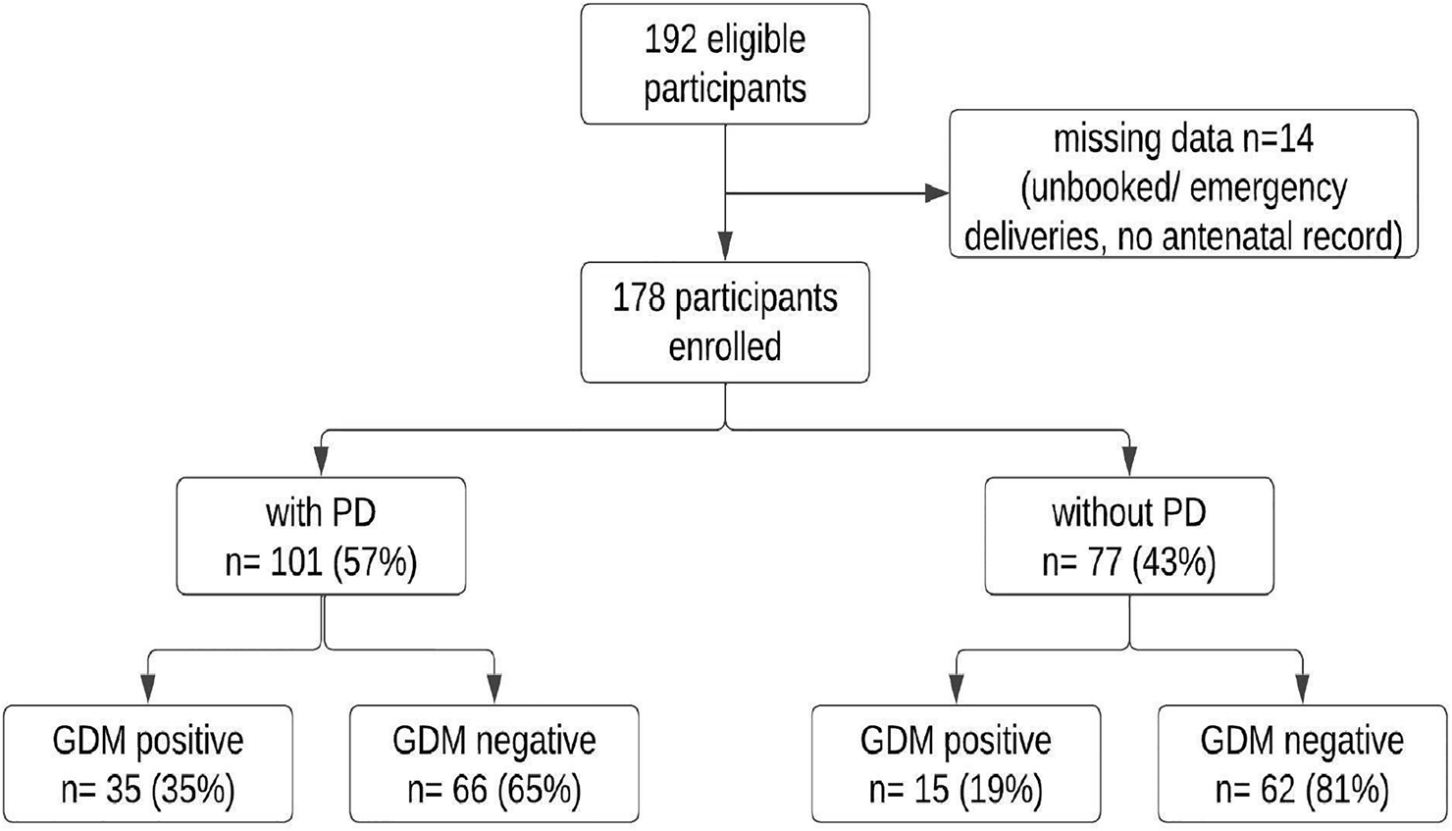
Study sample composition.

A total of 192 eligible women were recruited after an initial screening. Fourteen postpartum women were excluded due to lack of prior medical record, indicating un-booked or emergency cases of deliveries. Hence, a total of 178 postpartum women were evaluated in this study. Figure 1 shows the study flowchart. Out of 178 women, 50 (28%) had GDM. The mean age of women with GDM was 31.4 (± 5.2) years whereas those without GDM had a mean age of 29.2 (± 4.5) years (Table 1). Of those with GDM, majority were diagnosed in the third trimester (60%), followed by second (24%) and then first trimester (16%). Most of these (44%) were managed with oral medications only, followed by diet control only (28%), injectables (22%) and a combination of methods (6%). Majority of the women (72%) were fully active during their pregnancy. Among those who had GDM, 68% (n = 34) had a family history of diabetes. The GDM positive women were also found to have delivered male babies (60%) and gone through a C- Section (62%) for their delivery (Table 2). Upon clinical examination, majority (70%) of the GDM positive women exhibited periodontal disease (gingivitis or periodontitis, *p* value 0.03) (Table 3). A significant difference was found among women with GDM and those without GDM for variables such as age, existing comorbidity and family history of diabetes (Tables 1–2).

**Table 1:** Baseline characteristics and Sociodemographic factors of postpartum women in Aga Khan hospital, Karachi.

	Total postpartum women n (%)	Women with GDM n (%)	Women without GDM n (%)	P value
Age in years				
Mean (SD)	29.8 (4.8)	31.4 (5.2)	29.2 (4.5)	0.01*
BMI (kg/m^2^)				
Normal	79 (44.4)	14 (28)	65 (50.8)	0.004^†^
Overweight	58 (32.6)	17 (34)	41 (32)	
Obese	41 (23)	19 (38)	22 (17.2)	
Education				
College or less	37 (20.8)	12 (24)	25 (19.5)	0.17^†^
Graduate	84 (47.2)	18 (36)	66 (51.5)	
Postgraduate	57 (32)	20 (40)	37 (28.9)	
Employment				
Employed	50 (28.1)	13 (26)	37 (28.9)	0.70^†^
Housewives	128 (71.9)	37 (74)	91 (71.1)	
Ward Type				
General/semi	62 (34.8)	17 (34)	45 (35.2)	0.88^†^
Private	116 (65.2)	33 (66)	83 (64.8)	
Consanguineous marriage				
Yes	32 (18)	12 (24)	20 (15.6)	0.19^†^
No	146 (82)	38 (76)	108 (84.4)	
Existing comorbid				
Yes	55 (30.9)	21 (42)	34 (26.6)	0.04^†^
No	123 (69.1)	29 (58)	94 (73.4)	

^†^: Pearson chi square, *: t-test.

**Table 2:** Gestational factors of postpartum women in Aga Khan hospital, Karachi.

	Total postpartum women n (%)	Women with GDM n (%)	Women without GDM n (%)	P value
Physical activity during pregnancy				
Bedrest	12 (6.7)	4 (8)	8 (6.2)	0.89†
Fairly active	38 (21.4)	10 (20)	28 (21.9)	
Fully active	128 (71.9)	36 (72)	92 (71.9)	
Parity				
One	76 (42.7)	21 (42)	55 (43)	0.47†
Two	52 (29.2)	12 (24)	40 (31.2)	
Three or more	50 (28)	17 (34)	33 (25.8)	
Family history of DM				
Yes	100 (56.2)	34 (68)	66 (51.6)	0.04^†^
No	78 (43.8)	16 (32)	62 (48.4)	
Baby gender				
Female	90 (50.6)	20 (40)	70 (54.7)	0.08^†^
Male	88 (49.4)	30 (60)	58 (45.3)	
Baby weight				
Low birth weight	27 (15.2)	6 (12)	21 (16.4)	0.46^†^
Normal range	151 (84.8)	44 (88)	107 (83.6)	
Gestational age				
Preterm	40 (22.5)	16 (32)	24 (18.7)	0.06^†^
Term	138 (77.5)	34 (68)	104 (81.3)	
Mode of Delivery				
C-section	94 (52.8)	31 (62)	63 (49.2)	0.12^†^
Vaginal	84 (47.2)	19 (38)	65 (50.8)	

^†^: Pearson chi square, *: t-test.

**Table 3:** Dental factors of postpartum women in Aga Khan hospital, Karachi.

Dental characteristics		Total postpartum women n (%)	Women with GDM n (%)	Women without GDM n (%)	P value
Last dental visit	Never	88 (49.4)	24 (48)	64 (50)	0.55
	During pregnancy	29 (16.3)	8 (16)	21 (16.4)	
	1–2 years ago	45 (25.3)	13 (26)	32 (25.)	
	> 2 years ago	16 (9)	5 (10)	11 (8.6)	
Bleeding gums (as per participant)	Yes	80 (45)	23 (46)	57 (44.5)	0.86
	No	98 (55)	27 (54)	71 (55.5)	
Gingival index score	No gingivitis	77 (43.3)	15 (30)	62 (48.4)	0.007
	Mild gingivitis (0.1 -1)	45 (25.3)	11 (22)	34 (26.6)	
	moderate gingivitis (1.1–2)	56 (31.5)	24 (48)	32 (25)	
Periodontitis	Yes (≥ 4 teeth having ≥ 1 sites with PD ≥ 4 mm and CAL)	37 (20.8)	16 (32)	21 (16.4)	0.02
	No	141 (79.2)	34 (68)	107 (83.6)	
Periodontal disease	Gingivitis or periodontist	101 (56.7)	35 (70)	66 (48.4)	0.03
	No	77 (43.6)	15 (30)	62 (51.6)	

The multivariable analysis using cox proportional hazard ratio indicated presence of periodontal disease and BMI (obesity) as a predictor of GDM. The prevalence ratio of GDM among those with periodontal disease was 1.7 times greater (*p* value 0.07, 95% CI: 0.9–3.2) to those who did not have periodontal disease. However, this association turned out to be statistically insignificant. Statistically significant association was found between the prevalence ratio of GDM in women with obesity which was 2.6 times that of non-obese women (*p* value < 0.01, 95% CI: 1.3–5.1) keeping all other variables constant (Table 4). Multi-collinearity analysis showed that all the independent variables inserted in the regression models showed values of tolerance greater than 0.10 and Variance Inflation Factor (VIF) < 2. There was no statistical interaction or confounding observed among the variables as tested by adjusted regression model.

**Table 4:** Adjusted and unadjusted prevalence ratio with 95% CI from cox proportional regression model of postpartum women in Aga Khan hospital, Karachi.

	Unadjusted prevalence ratio (95% CI)	Adjusted prevalence ratio (95% CI)
Age (years)	1.1 (1.01–1.12)	–
BMI (kg/m^2^)		
Over weight	1.6 (0.82–3.35)	1.6 (0.78–3.21)
Obese	2.6 (1.31–5.22)	2.6 (1.29–5.14)
Family history of DM	1.7 (0.91–3.00)	–
Other illness (comorbid)	1.6 (0.92–2.84)	–
Baby gender (male)	1.5 (0.87–2.71)	–
Gestational age (preterm)	1.6 (0.90–2.90)	–
Delivery Type (C-section)	1.5 (0.82–2.59)	–
Periodontal disease	1.8 (0.97–3.26)	1.7 (0.95–3.20)

## Discussion

The main finding of this study suggests that the prevalence ratio of GDM in women with periodontal disease is 1.7 times as compared to women without periodontal disease, however the association is statistically insignificant. A statistically significant association was found between the prevalence ratio of GDM in women with obesity. It was 2.6 times compared to women who were not obese.

There is evident biological plausibility of periodontal disease contributing to systemic spread of bacteria and bacterial products inducing a systemic inflammatory response that can lead to numerous diseases including GDM^30^. The role of inflammation in the pathogenesis of diabetes justifies the interest in investigating the association between the two conditions. The effect of inflammatory mediators such as IL-6, CRP and TNF-α on glucose metabolism can influence an antagonistic action on insulin^31^. Based on this hypothesis of association, it has been speculated that periodontal disease contributes to the development of insulin resistance observed in women with GDM. Current scientific evidence points out the bidirectional relationship of the conditions whereby DM is associated with an increase in progression and incidence of periodontal disease while periodontal disease is reported to worsen glycaemic control seen in DM patients^32^. However, GDM has a multifactorial origin with other reported risk factors including age, education, socioeconomic status, pre-pregnancy BMI/ obesity, ethnicity, family history of diabetes, history of GDM, high parity, short stature and polycystic ovary syndrome (PCOS)^33–41^.

A systematic review conducted by Samuel A. and Brian W. found periodontitis to be significantly associated with increased risk for GDM compared to women without periodontitis^42^. Despite the statistical significance of the results, the study highlights the lack of robust study designs to substantiate the findings. Also, just like findings from our study, 2 out of the 3 cross sectional studies included in the systematic review reported insignificant association between periodontitis and GDM. Another systematic review of epidemiologic observational studies reported the evidence of adverse effects of periodontal disease on diabetes outcomes^43^. The study similar to ours, also concluded the need of further longitudinal studies with bigger sample sizes and greater generalizability.

The lack of scientific evidence to affirm a positive association between periodontal disease and GDM was reported by Esteves et al*.* in a meta-analysis^44^. One of the main reasons pointed out was the lack of clinical heterogeneity whereby studies do not follow the same criteria to define periodontal disease and non-uniform methods for diagnosing GDM status. Another study reported no association between PD and GDM with a p value = 0.053, despite having participants from similar education and status background^6^. Comparable to our study, Novak et al. concluded insignificant association between the conditions (OR = 2, 95%CI = 0.6–6.3) and in models adjusting for potential confounders^45^.

Obesity is an important risk factor of GDM. The conditions together can result in adverse birth outcomes^46^. Obesity is reported to be positively associated with clustering of metabolic risk factors of GDM (p value < 0.05)^47^. Close to our findings, a meta-analysis reported odds of developing GDM among obese women to be 3.56 (95% CI: 3.05–4.21) times higher^48^. A few studies have examined the association between pregnancy obesity and periodontal disease among diabetic pregnant women. A cohort study reported no difference in association of obesity and periodontal disease among women with gestational diabetes and those without gestational diabetes^49^. Another study reported severity in periodontal diseases among women with GDM and obesity as compared to women who were normal weight ^50^. This variation of findings suggests a further need to probe the relationship of these conditions together. Our study also reported a significant difference in variables such as age, existing comorbidity and family history of diabetes in women with GDM and those without GDM. Studies have reported similar findings and also other risk factors of GDM including socioeconomic status, education, parity, consanguineous marriage, physical activity and lifestyle habits^7,11,18,34,36,41,48,49^.

### Study strengths and limitations

The generalizability of this study is slightly limited as this study is a single center study conducted at a private tertiary care hospital. Despite Aga Khan hospital, stadium road, Karachi serves about 500 deliveries per month with a mixture of population from all sorts of backgrounds and socioeconomic status, however, it cannot be considered a representative of the general population of women with a potential bias of higher medical risk or socioeconomic status and proper care given throughout the antenatal period to all pregnant women. The level of care given during the antenatal period reflects in our findings of low adverse pregnancy outcomes. Misclassification bias may have occurred as there a lack of uniformity in the guidelines for screening and diagnosing GDM^51^. However, this study uses validated standard methods to define both GDM and periodontal disease. The cross-sectional study design, limits the assessment of temporality of development of the conditions. Lack of pre-pregnancy maternal weight is also a limiting factor in assessing the relationship of the association as pre-pregnancy BMI is also a known potential confounder^38^. No tool was used to measure physical activity during pregnancy and the question was recall based as study population was postpartum women. To the best of our knowledge, this is a pioneer study from the region, with methodological rigor, evaluating postpartum women for association between PD and GDM.

### Public health implications and recommendations

Improving oral health and treating periodontal disease before and during pregnancy may reduce and perinatal morbidity associated with the conditions and prevent type 2 diabetes after pregnancy. Strategic planning is needed as part of antenatal program to improve dental health care and to reduce periodontal diseases among pregnant females and inclusion of dietary guidance for weight control in antennal care. The study calls attention of doctors and dentists to the importance of the transdisciplinary and holistic approach of the pregnant woman in order to offer prevention and treatment for these patients and, consequently, improve the health of their children.

Future studies should perform analyses evaluating glycaemic control and its relationship with inflammatory mediators in saliva and plasma. Some laboratory analyses at molecular levels are necessary to better understand the systemic diseases’ effect on periodontium.

## Conclusion

There is insignificant association found between the prevalence ratio of GDM in women with periodontal disease in our setting. Women who are overweight or tend to gain weight should be closely monitored and guided to take dietary measures. Timely measures can prevent both periodontal disease and subsequent GDM to avoid unfavourable outcomes, especially in low resource countries such as Pakistan where a dental check-up is not a part of regular antenatal care.

## Data Availability

The dataset used for analysis during the current study is available from the corresponding author on reasonable request.
